# REGA-SIGN: Development of a Novel Set of NanoBRET-Based G Protein Biosensors

**DOI:** 10.3390/bios13080767

**Published:** 2023-07-28

**Authors:** Katrijn Boon, Nathan Vanalken, Eef Meyen, Dominique Schols, Tom Van Loy

**Affiliations:** Laboratory of Virology and Chemotherapy, Department of Microbiology, Immunology and Transplantation, Rega Institute, KU Leuven, Herestraat 49, P.O. Box 1030, 3000 Leuven, Belgium; katrijn.boon@kuleuven.be (K.B.); nathan.vanalken@kuleuven.be (N.V.); eef.meyen@kuleuven.be (E.M.); dominique.schols@kuleuven.be (D.S.)

**Keywords:** NanoBRET, G protein activation, G protein-coupled receptor, biosensor, kinetic

## Abstract

Despite G protein-coupled receptors (GPCRs) being important theapeutic targets, the signaling properties of many GPCRs remain poorly characterized. GPCR activation primarily initiates heterotrimeric G protein signaling. To detect ligand-induced G protein activation, Bioluminescence Resonance Energy Transfer (BRET)-based biosensors were previously developed. Here, we designed a novel set of Nanoluciferase (NLuc) BRET-based biosensors (REGA-SIGN) that covers all Gα protein families (i.e., Gα_i/o_, Gα_Ss/L_, Gα_12/13_ and Gα_q/15_). REGA-SIGN uses NLuc as a bioluminescent donor and LSS-mKATE2, a red-shifted fluorophore, as an acceptor. Due to the enhanced spectral separation between donor and acceptor emission and the availability of a stable substrate for NLuc, this donor–acceptor pair enables sensitive kinetic assessment of G protein activity. After optimization, the NLuc integration sites into the Gα subunit largely corresponded with previously reported integration sites, except for Gα_Ss/L_ for which we describe an alternative NLuc insertion site. G protein rescue experiments validated the biological activity of these Gα donor proteins. Direct comparison between EGFP and LSS-mKATE2 as acceptor fluorophores revealed improved sensitivity for nearly all G protein subtypes when using the latter one. Hence, REGA-SIGN can be used as a panel of kinetic G protein biosensors with high sensitivity.

## 1. Introduction

G protein-coupled receptors (GPCRs) are the largest family of cell-surface proteins that are critically involved in regulating developmental and physiological functions by responding to a variety of extracellular messengers such as protein and peptide hormones, amines, ions, light and odorants. Deregulated GPCR signaling contributes to various human pathologies, including viral infections, cancer and metabolic and immunological disorders. Accordingly, GPCRs are the leading target for FDA-approved drugs [1,2,3].

GPCR activation by an extracellular ligand primarily initiates G protein-mediated signaling, which involves the activation of heterotrimeric Gαβγ proteins which are associated with the activated receptor via its intracellular loops [4]. Four main families of G proteins exist, based on the type of Gα subunit (i.e., Gα_i/O_, Gα_S_, Gα_q/15_ and Gα_12/13_). Receptor activation leads to a conformational change in the heterotrimer G protein complex, enabling the exchange of GDP for GTP at the Gα subunit and the dissociation (or reorganization) of the Gα-GTP and Gβγ subunits. Subsequently, both subunits activate downstream effector molecules like adenylate cyclase, phospholipase C (PLC), GTPases and Ca^2+^ channels [5]. Functional assays measuring GPCR activity are often based upon the direct or indirect detection of the activity of such effector molecules and their second messengers [6] with an assay readout that is often subject to signal amplification [7].

An alternative strategy to study GPCR activation is to directly assess G protein activity by proximity-based Bioluminescence Resonance Energy Transfer (BRET)-based assays. Hereby, a bioluminescent donor molecule is incorporated into the Gα subunit, and a fluorescent acceptor molecule is fused to the Gγ subunit. In an inactive receptor state, the Gα and Gβγ subunits of the heterotrimeric G protein signaling complex are in close proximity allowing the donor molecule to excite the acceptor fluorophore thereby generating a BRET signal. GPCR activation, and the resulting G protein dissociation, increases the distance between the Gα and Gβγ subunits leading to reduced acceptor fluorophore activation. BRET-based biosensors thus exploit the change in relative orientation and distance between the activated Gα subunit and Gβγ subunits to directly detect and quantify GPCR-mediated G protein activation [8,9,10,11].

Several generations of BRET-based G protein assays have been developed using various donor and acceptor fluorophore combinations, as well as different donor substrates. BRET1, the original BRET technology, uses Renilla Luciferase (RLuc) as the donor protein combined with coelenterazine-h as a substrate to generate bioluminescence which excites a yellow fluorescent acceptor protein like YFP. This substrate has a long lifetime, and BRET1 is characterized by robust BRET signals. Nonetheless, the donor and acceptor emission spectra overlap significantly, resulting in the bleed-through of the donor signal into the acceptor signal, which leads to a low signal-to-noise ratio of the BRET readout. To reduce the spectral overlap between donor and acceptor emission, BRET2 uses a novel substrate (Coelenterazine 400a or DeepBlueC) to lower the emission peak of RLuc and combines this with appropriate acceptor fluorophores like the green fluorescent proteins EGFP or GFP10. However, this change in substrate results in low light emission and a short lifetime of the RLuc bioluminescence, reducing the sensitivity and measurement time. Eventually, eBRET2 (enhanced BRET2) introduced the modified RLuc8 which resulted in a 5–30-fold enhancement of the original BRET2 signal, resolving the low light emission but still suffering from the short lifetime of the substrate [12]. Recently, a new luciferase enzyme (NanoLuciferase, NLuc), originally derived from a naturally occurring luciferase in the deep sea shrimp *O. gracilirostris*, was developed [13]. NLuc has greatly enhanced luminescence and uses a substrate with improved stability, enabling kinetic measurements. Because of its brightness, NLuc can be paired with a red-shifted fluorophore, which increases the spectral separation between donor and acceptor emission and improves the signal-to-noise ratio when compared to BRET1 and eBRET2 pairs [14,15,16]. Also, given its relatively small size (19 kDa), it is less prone to alter the biological function and interactions of the protein in which it is inserted [13]. NLuc-related BRET assays are referred to as NanoBRET.

BRET1, eBRET2 and NanoBRET were previously used to make BRET-based G protein biosensor assays [8,9,10,11,17,18]. Not all BRET-based biosensor packages, however, support extended kinetic read modes, despite the fact that insight into the kinetics and dynamics of responses could be of added value in quantitative pharmacology [19,20]. Hence, we aimed to develop and validate a novel set of NanoBRET-based G protein biosensors that have the ability to kinetically detect G protein activity with large signal-to-noise separation. This new panel of biosensors was designated REGA-SIGN (REaltime G protein Activation SIGNaling). REGA-SIGN is, to our knowledge, the first G protein biosensor to use NLuc in combination with a red-shifted fluorophore. It can be used to directly evaluate G protein activation across all G protein families using a kinetic read mode while ensuring high spectral separation between the BRET pair.

## 2. Materials and Methods

### 2.1. Plasmids, Reagents and Compounds

pBABE-puro-NLS-LSSmKate2 was a gift from Vladislav Verkhusha (Addgene plasmid #34586). Gα_i1_ (#GNAI100000), Gα_i2_ (#GNAI200000), Gα_i3_ (#GNAI300000), Gα_OA_ (#GNA0OA0000), Gα_OB_ (#GNA0OB0000), Gα_Ss_ (#GNA0SS0000), Gα_SL_ (#GNA0SL0000), Gα_12_ (#GNA1200000), Gα_13_ (#GNA1300001), Gα_q_ (#GNA0Q00000), Gα_15_ (#GNA1500000) Gγ1 (#GNG0100000), Gγ2 (#GNG0200000), Gγ9 (#GNG0900000), Gβ1 (#GNB0100000), β2-adrenergic receptor (#AR0B200000), Thromboxane A2 receptor (#TXA2R00000) and Histamine 3 receptor (#HRH0300000) were purchased from cDNA Resource Centre and are in the pcDNA3.1(+) backbone. The pNLF1-N vector (#N1351) and pGlosensor-22F cAMP plasmid (#E2301) were purchased from Promega. Pertussis toxin (PTX; #3097) was purchased from Tocris, Bristol, UK, Cholera Toxin (CTX; #C8052) from Merck, Rahway, NJ, USA and YM-254890 (#257-00631) from FUIJIFILM Wako Chemicals, Osaka, Japan. Histamine dihydrochloride (Histamine; #3545) was purchased from Bio-Techne, Minneapolis, MN, USA; Isoprenaline hydrochloride (Isoprenaline; #I5627), 9,11-Dideoxy-11α,9α-epoxymethanoprostaglandin F2α (U46619; #D8174), 3-isobutyl-1-methylxanthine (IBMX; #I7018), Forskolin (FSK; #F6886) and Poly-D-Lysine (PDL; #2780) from MERCK. Nano-Glo^®^ Vivazine™ substrate (#N2581) and NanoBRET™ Nano-Glo^®^ Substrate (#N1571) were purchased from Promega, Madison, WI, USA.

### 2.2. Cloning of G Protein Biosensor Plasmids

To construct the Gγ-LSS-mKATE2(N) plasmids, PCR was used to linearize pcDNA3.1(+)Gγ and to add a sequence complementary with the LSS-mKATE2 fragment to the Gγ N-terminus. The LSS-mKATE2 fragment flanked with sequences complementary to the Gγ gene was created via PCR, using pBABE-puro-NLS-LSSmKate2 as a template. Eventually, HiFi DNA assembly mastermix (New England Biolabs, Ipswich, MA, USA, E2621S) was used to generate the N-terminally tagged (LSS-mKATE2) Gγ-protein plasmids. This set-up was repeated for the EGFP-tagged Gγ proteins. Gα-NLuc expression plasmids were generated by similar methods. Here, a single backbone template for each insertion site within the Gα protein was linearized, and complementary sequences for the NanoLuciferase (NLuc) flanked by an SGGGS linker at both ends were added. During PCR amplification of the NLuc cDNA, the pNLF1-N vector was used as a template, and a flexible SGGGS linker was added on both sides via PCR. Using HiFi DNA assembly mastermix (New England Biolabs, E2621S), the SGGGS flanked NLuc was finally inserted in the linearized pcDNA3.1(+)Gα backbone generating the Gα-NLuc constructs. All plasmids were verified by DNA sequencing.

### 2.3. Cell Lines

HEK293A knock-out (KO) cells (Table 1) were kindly provided by Dr. A. Inoue (Tohoku University, Sendai, Japan) [21,22,23]. Wild-type HEK293T cells, as well as the KO cell lines, were cultured in Dulbecco’s Modified Eagle Medium, high glucose (DMEM; #41965, Thermo Fisher Scientific, Waltham, MA, USA) supplemented with 10% Fetal Bovine Serum (FBS; #10270106, Thermo Fisher Scientific), from here on referred to as growth medium.

### 2.4. NanoBRET-Based G Protein Activation Assay

HEK293T.WT cells were transiently co-transfected in suspension with a model receptor, Gα-NLuc donor plasmid and untagged Gγ, Gγ-LSS-mKATE2(N) or Gγ-EGFP(N) acceptor plasmid in a 1:1:10 ratio (with a total of 1 µg/µL DNA), respectively. For testing the effect of additional co-transfection of Gβ1 subunit, cells were transiently co-transfected in suspension with H3R, Gα_i1_-91-NLuc, Gβ1 (or transfection carrier DNA) and Gγ-LSS-mKATE2(N), respectively, in a 1:1:1:10 ratio. For all transfections, FuGENE^®^ HD Transfection Reagent (#E2311; Promega) was used at a 3:1 reagent to DNA ratio. The FuGENE^®^ HD/DNA mixture was incubated for 10 min at ambient temperature before adding it to the cell suspension.

Transfected cells were seeded at a density of 3.0 × 10^4^ cells/well in white, clear flat-bottom 96-well plates pre-coated with 100 µg/mL poly-D-lysin and incubated for 48 h at 37 °C and 5% CO_2_. In case of PTX or CTX addition, 25 µL of 5X concentrated compound (final concentration of 50 ng/µL and 10 µM, respectively) was added after 24 h of incubation. Forty-eight hours after transfection and seeding, cells were washed with assay buffer (Hank’s Balanced Salt Solution (HBSS; Thermo Fisher Scientific), 20 mM HEPES buffer (Thermo Fischer Scientific), 0.5% FBS) and incubated with 90 µL of a 1:100 Nano-Glo^®^ Vivazine™ working solution (#N2581, Promega) for 45 min at 37 °C and 5% CO_2_. If inhibitor YM-254890 was added, it was dissolved in the Vivazine solution at a concentration of 1 µM. Plates were transferred to the FLIPR Tetra (Molecular Devices, Silicon Valley, CA, USA). After 15 min of equilibration time, baseline BRET signals (i.e., five consecutive measurements of donor and acceptor emission) were measured immediately followed by the automatic addition of 10 µL of 10X ligand to the cell plate with the FLIPR Tetra. Changes in BRET signal were monitored in real time for 25 min every 2.5 s. Measurements were performed using a 440–480 nm donor emission filter (#0200-6179, Molecular Devices) and a custom 615 nm AT600lp LSS-mKATE2 acceptor emission filter (#296420, Chroma, Irvine, CA, USA) or 515–575 nm EGFP acceptor emission filter (#0200-6203, Molecular Devices) [24].

### 2.5. Determination of Acceptor Fluorophore Expression by Flow Cytometry

HEK293T.WT cells were transiently co-transfected in suspension with Gα_i1_ donor plasmid, Gγ-LSS-mKATE2(N) acceptor plasmid and, in case of addition of Gβ1, also with the β1 plasmid (or transfection carrier DNA). Cells were seeded as described before. After 48 h, cells were detached using 0.25% Trypsin-EDTA (Thermo fisher Scientific, #25200056) and resuspended in assay buffer. Samples were analyzed for acceptor fluorophore expression by flow cytometry (BD FACSCelesta^TM^ HTS, BD Bioscience, San Jose, CA, USA). Data were further analyzed using FlowJo^TM^ software (Ashland, OR, USA). Mean fluorescent intensity (MFI) was taken as measure for LSS-mKATE2 protein expression. For statistical analysis, Tukey’s multiple-comparison test or unpaired *t*-test was used in GraphPadV9.3.1.

### 2.6. Donor Saturation Assay (DSA)

DSA experiments were performed as described by Promega [25]. Briefly, HEK293T.WT cells were transiently co-transfected in suspension and seeded as described before with the optimized donor–acceptor plasmid pairs. For transfection, a fixed amount of Gα-NLuc donor plasmid (100 ng) but increasing amounts of Gγ-LSS-mKATE2 acceptor plasmids were used to obtain increasing acceptor-to-donor plasmid ratios. Therefore, Gγ-LSS-mKATE2 plasmid (max. 1000 ng, corresponding to the ratio used in the NanoBRET assay) was serially diluted (1:3) into Transfection carrier DNA (#E4881; Promega). As a negative control, only Transfection carrier DNA (#E4881; Promega) was combined with the Gα-NLuc donor (100 ng). Forty-eight hours post transfection, cells were washed with assay buffer, and subsequently, 100 µL of a 1:500 NanoBRET™ Nano-Glo^®^ Substrate (#N1571, Promega) was added to the plate. Immediately upon substrate admission, the plate was transferred to the FLIPR Tetra, and 15 consecutive reads were obtained as the end-point measurement using a 440–480 nm donor emission filter (#0200-6179, Molecular Devices) and a custom 615 nm AT600lp acceptor emission filter (#296420, Chroma). Afterward, BRET ratios were plotted in GraphPad against the transfected acceptor-to-donor plasmid ratio to determine the shape of the curve.

### 2.7. cAMP Inhibition Gα_i/o_ Rescue Experiment

HEK293-ΔGαi cells were transfected in suspension with Histamine 3 receptor (H3R) plasmid, pGlosensor-22F (#E2301, Promega) and either a Gα_i/O_-NLuc donor plasmid (rescue), wild-type Gα_i-_protein plasmid (PC) or Transfection carrier DNA (#E4881; Promega) (NC). Transfected cells were seeded at a density of 3 × 10^4^ cells/well in white 96-well plates with clear flat bottom (#CLS3610, Merck) pre-coated with 100 µg/mL poly-D-lysin (#2780, Merck). Cells were incubated for 48 h at 37 °C and 5% CO2, after which they were washed with CO_2_-independent medium (#18045-054, Thermo Fisher Scientific) supplemented with 10% FBS. CO_2_-independent medium/10% FBS supplemented with 300 µM 3-isobutyl-1-methylxanthine (#I7018, MERCK) and 2% GloSensor cAMP reagent (#E1291, Promega) was then added to the cells (100 µL/well). After 2 h of incubation at 37 °C, baseline luminescence was measured every 5 s for 30 s using the FLIPR Penta. Thereafter, 25 µL of 5X Histamine (final concentration of 1µM) was automatically added to the cell plate, and bioluminescence was monitored in real time every 5 s for 10 min. Next, 25 µL Forskolin (#F6886, Merck) was added at a final concentration of 5 µM, and bioluminescence was monitored in real time for 40 min every 5 s. Statistical analysis was accomplished with one-way ANOVA followed by Dunnett’s test.

### 2.8. cAMP Production Gα_S_ Rescue Experiment

HEK293-ΔGαs cells were transfected in suspension with β2-adrenergic receptor (B2AR), pGlosensor-22F and either a Gα_S_-NLuc plasmid (rescue), untagged Gα_S_-protein plasmid (PC) or transfection carrier DNA (#E4881; Promega) (NC). Transfected cells were seeded and washed, and baseline luminescence was measured as described above for the cAMP inhibition assay. Thereafter, 25 µL of 5X Isoprenaline (final concentration of 10 µM) was added to the cell plate with the FLIPR Penta, and changes in bioluminescence were monitored in real time for 10 min every 5 s. Statistical analysis was performed with one-way ANOVA followed by Dunnett’s test.

### 2.9. Ca^2+^-Signaling Gα_q_ Rescue Experiment

HEK293-ΔGα_q_ cells were transfected in suspension with Thromboxane A2 receptor (TBX) and either a Gα_q_-91-NLuc donor plasmid, untagged Gα_q_-protein plasmid (PC) or transfection carrier DNA (#E4881; Promega) (NC). Transfected cells were seeded at a density of 3.0 × 10^4^ cells/well in black, clear flat-bottom 96-well plates (#CLS3610, Merck) coated with 100 µg/mL poly-D-lysin (#2780, Merck) and incubated for 48 h at 37 °C and 5% CO_2_. Subsequently, the cells were loaded with 80 µL Calcium 6-dye mix containing 50% of FLIPR calcium 6-dye (Ex: 485 nm, Em:525 nm; #R8290; Molecular Devices) and 50% assay buffer. After dye administration, the cells were incubated for two hours at 37 °C and 5% CO_2_. The plate was transferred to the FLIPR Tetra (Molecular Devices), and baseline fluorescence was measured with 5 consecutive reads. Afterward, 20 µL of 5X U46619 (#D8174; MERCK) was added to obtain a final concentration of 1 µM. Changes in cytosolic calcium concentrations were measured in all 96 wells simultaneously with the FLIPR tetra. For each sample, the response over baseline after addition of U46619 was calculated and background-corrected using the ScreenWorks 4.0TM software (Molecular Devices). The maximum response induced by transfection of PC was set at 100%, and data were normalized to this value (GraphPad Software, San Diego, CA, USA). Statistical analysis was accomplished with one-way ANOVA followed by Dunnett’s test.

### 2.10. BRET Data Analysis

G protein activation using the NanoBRET assay was calculated using the equations presented in Calculation 1, which are graphically presented in Figure S1 of Supplementary Material. BRET ratios were calculated by taking the ratio of LSS-mKATE2 acceptor emission (615 nm) to NLuc donor emission (440–480 nm). The basal BRET ratio (BRET_basal_) was defined as the mean BRET ratio of the five consecutive readings prior to ligand addition. To quantify ligand-induced changes, ∆BRET was calculated for each well as the % difference to baseline. Subsequently, ∆BRET values were background-corrected by subtracting the average ∆BRET of the negative (vehicle) control wells. Finally, the negative area under the curve (neg AUC) was used as the readout for G protein activation. Dose–response curves were fitted to *log(agonist)* vs. *response—Find ECanything* model in GraphPad V9.3.1 (GraphPad Software, San Diego, CA, USA) whereby the hillslope, bottom and F values were constrained to 1, 0 and 50, respectively. The calculated top value was taken as E_max_. The analysis was accomplished with one-way ANOVA followed by Dunnett’s test.

**Calculation 1.** BRET signal calculation.

1. BRET ratio = $\frac{615nm_{em}}{460nm_{em}}$
2. ∆BRET = $\left[\frac{BRET_{stim}-BRET_{basal}}{BRET_{basal}}\right]\times 100$
3. NC_corrected_∆BRET = ∆BRET_exp_—mean∆BRET_NC_

## 3. Results

### 3.1. Characterization of the New Donor–Acceptor NanoBRET Pair Kinetics

BRET-based G protein biosensors previously showed their value for the detection and analysis of receptor-mediated heterotrimeric G protein activation in living cells [8,9,10,11,12,14,17,26,27]. Most of these biosensors are BRET1- or eBRET2-based assays using Renilla luciferase (RLuc or RLuc8, respectively) [12,14] as a bioluminescent donor paired with a yellow- or green-shifted acceptor fluorophore [26]. In this study, we aimed to develop a highly sensitive kinetic biosensor by combining Nanoluciferase (NLuc) with LSS-mKATE2, a red-shifted fluorophore with a long Stokes shift [28]. The new NanoBRET-based G protein-biosensor package, designated as REGA-SIGN, includes eleven different biosensors (Gα_i1_, Gα_i2_, Gα_i3_, Gα_OA_, Gα_OB_, Gα_Ss_, Gα_SL_, Gα_12_, Gα_13_, Gα_q_ and Gα_15_), thereby covering the four major classes of G proteins. The sensitivity of BRET-based G protein biosensors depends on various factors including the BRET-pair characteristics, the co-expression of tagged and untagged G proteins and the Gα-subunit donor insertion site and its paired Gγ subtype [8,17,25]. For the initial characterization and optimization of REGA-SIGN, we opted to use Gα_i1_ and Gγ2 as the model G protein biosensor pair, since it is one of the most thoroughly described [8,18]. Hence, we used its commonly accepted donor insertion site (L(91)K) and coupled Gγ2 protein to generate the biosensor pair Gα_i1_-91-NLuc/Gγ2-LSS-mKATE2.

**Figure 1:**
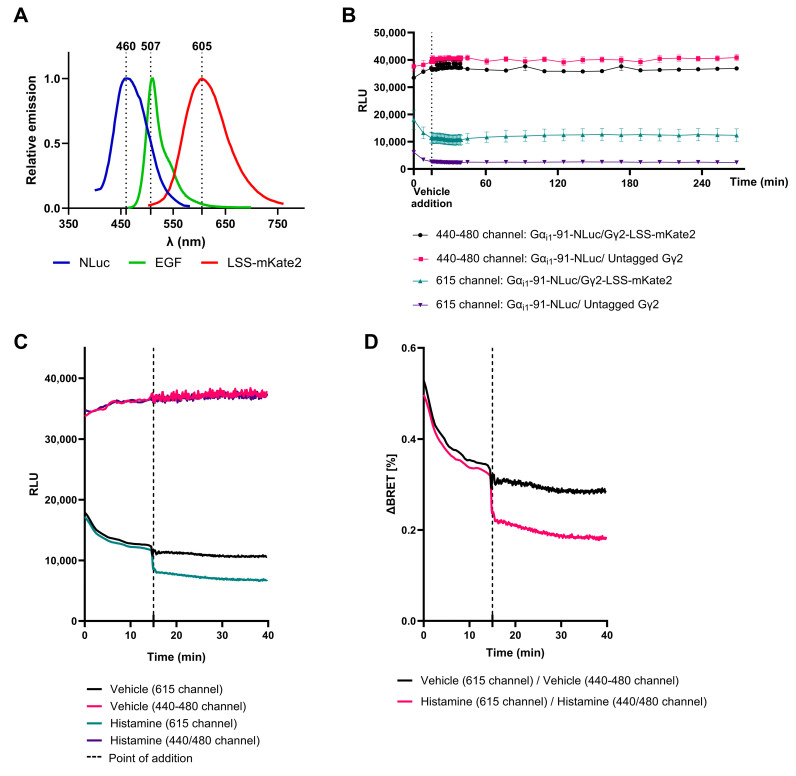
BRET-pair characteristics. (**A**) Graphical representation of spectral properties [29] of EGFP and LSS-mKATE2 combined with NLuc. (**B**) Luminescent signals in the 440–480 nm channel (energy donor emission filter) and 615 nm channel (energy acceptor emission filter) were measured before and after vehicle addition to HEK293T.WT cells co-transfected with the Histamine 3 receptor (H3R), Gα_i1_-91-NLuc and either untagged Gγ2 or tagged Gγ2-LSS-mKATE2 and equilibrated with Vivazine substrate. Data show the mean ± SEM of three independent experiments. For clarity, the shown data points were limited by using *Graph faster—skip 15 data points* in GraphPad V9.3.1. (**C**,**D**) Time-course experiment of HEK293T.WT cells co-transfected with H3R, Gα_i1_-91-NLuc and Gγ2-LSS-mKATE2 stimulated with 1 µM Histamine or vehicle control. Luminescence signals in the donor and acceptor channel are shown (**C**) and were used to calculate the depicted BRET ratio (**D**). Data show the mean ± SEM of three independent experiments which for clarity were smoothed using GraphPad V9.3.1.

We selected LSS-mKATE2 after comparing its spectral properties with the properties of the commonly used EGFP. Despite being less bright, the increased spectral separation between NLuc and LSS-mKATE2 emission (145 nm) was hypothesized to be advantageous for an improved signal-to-noise ratio (Figure 1A) [29]. The first step was then to investigate the BRET-pair characteristics including the donor bioluminescence stability, donor bioluminescence contamination in the acceptor channel (615 nm channel) and the ability to detect the LSS-mKATE2 fluorescent signal in the 615 channel. Therefore, HEK293T.WT cells were co-transfected with the Histamine 3 receptor (H3R; known signaling activation of Gα_i1_), Gα_i1_-91-NLuc and either untagged Gγ2 or Gγ2-LSS-mKATE2. Forty-eight hours post transfection, the donor substrate (Vivazine, Promega) was added. After substrate incubation, the luminescent signals were measured in both the 440–480 nm channel and 615 nm channel. As can be seen in Figure 1B, the donor signal remains stable over an extended period of time, and there is little contamination of this donor luminescence in the 615 nm channel. The acceptor fluorescent signal is also sufficiently bright to be detected and easily distinguished from the donor signal contamination in the 615 nm channel. These data ensure the REGA-SIGN donor–acceptor pair has a stable donor signal even after vehicle addition [14,15]. Next, we performed a similar time-course experiment to characterize the kinetics of receptor activation. Here, we used HEK293T.WT cells co-transfected with H3R, Gα_i1_-91-NLuc and Gγ2-LSS-mKATE2, which were then treated with histamine (1 µM) or a vehicle. After the addition of the vehicle control or histamine, the donor signal remained stable. In contrast, histamine addition resulted in an immediate drop in acceptor emission that stayed low over an extended period of time. Adding a vehicle only did not induce this effect (Figure 1C). In the case of histamine stimulation, these donor and acceptor emission profiles translated into a fast and stable drop in the BRET ratio (Figure 1D), for which we opted to take the Neg AUC as a readout (Calculation 1, Figure S1).

### 3.2. Necessity of the β1 Co-Transfection

**Figure 2:**
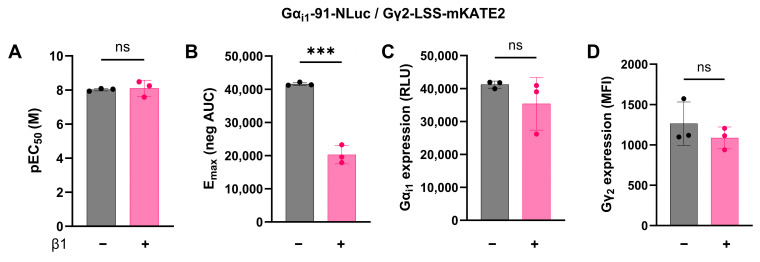
Impact of Gβ1 co-transfection on biosensor sensitivity. HEK293T.WT cells were co-transfected with the H3R, Gα_i1_-91-NLuc, Gγ2-LSS-mKATE2 and either untagged β1 (+) or transfection carrier DNA (-). Forty-eight hours post transfection, donor substrate (Vivazine, Promega) was added, and cells were stimulated with a 1:3 dilution series of Histamine (1 µM). BRET ratios were measured for 25 min after receptor stimulation. Subsequently, the dose–response curves were fitted to *log(agonist)* vs. *response—Find ECanything model* in GraphPad V9.3.1 (GraphPad Software, San Diego, CA, USA) and were used to calculate the pEC50 (**A**) and the top values (E_max_) (**B**). Luminescent donor emission signals before ligand stimulation were used as a surrogate for the donor expression levels (**C**). (**D**) For each transfection, the mean fluorescent intensity (MFI) of the total LSS-mKATE2 fluorescence was determined by flow cytometry. This serves as a surrogate for the acceptor expression levels. Data represent the mean of three independent transfections ± SEM. Data were tested by using an unpaired *t*-test (non-significant (ns); *** *p* < 0.001).

In several types of GPCR and G protein biosensor approaches, it was previously established that the co-transfection of untagged Gβ1 protein ensures the formation of functional heterotrimeric G protein complexes [8,9,10,11,17,26,27]. However, recently, it has been shown that overexpressed tagged Gα proteins were able to form functional G proteins by associating with endogenously expressed Gβγ proteins [30] thereby challenging the need for their co-transfection. Accordingly, we aimed to investigate the impact of Gβ1 co-expression in our biosensor approach. For this, HEK293T.WT cells were transiently co-transfected with H3R, Gα_i1_-91-NLuc, Gγ2-LSS-mKATE and either untagged Gβ1 or transfection carrier DNA. Forty-eight hours post transfection, cells were incubated with Vivazine, and following substrate stabilization, a dilution series of histamine was automatically added. Dose-dependent histamine responses were measured using the negative area under the curve (Neg AUC) as the readout for G protein activation (Figure S1, Calculation 1). pEC50 and E_max_ values were subsequently calculated from these data by using non-linear curve fitting. We found that the co-transfection of Gβ1 did not significantly affect the pEC50 value for receptor activation (Figure 2A), whereas the E_max_ value without Gβ1 co-transfection was significantly higher (Figure 2B). An increased E_max_ directly translates to a larger signal window and therefore a more sensitive assay.

To ensure this observation was not caused by differential protein expression, we quantified the expression level of tagged Gα and Gγ proteins in both conditions using, respectively, the basal luminescent signal and the mean fluorescent intensity (MFI, detected using flow cytometry). Gα_i1_-91-NLuc luminescence, as well as the total Gγ2-LSS-mKATE2 fluorescence, was not significantly different between both conditions (Figure 2C,D), thereby excluding differential protein expression. Hence, for the further development and optimization of REGA-SIGN, we opted to only co-transfect the tagged G proteins with the receptor of interest.

### 3.3. Determination of the Most Optimal Donor–Acceptor Pairs for REGA-SIGN

As mentioned, the sensitivity of BRET-based G protein biosensors also depends on the position at which the donor molecule is inserted into the Gα subunit, as well as its paired Gγ subtype [8,17,25]. Therefore, the next step in optimizing REGA-SIGN was to generate a set of donor plasmids that differed in the NLuc integration site within the respective Gα subunits. By further combining all of these donor plasmids with the different acceptor plasmids, we then aimed to determine which donor–acceptor pairs yielded the most pronounced BRET signals upon receptor stimulation. Previously reported BRET-based G protein biosensor studies were exploited to select one or two of the most commonly used donor insertion sites for each of the eleven Gα subunits and to identify the Gγ subtypes they preferentially paired with [8,9,11,17]. All of these donor insertion sites were found in the flexible loops between the α-helices of the Gα subunit, except for Gα_15_ for which the switch III region was recently identified as a novel donor insertion site [8]. The Gα-NLuc variants with these differential insertion sites were generated. Given that the Gγ1, Gγ2 and Gγ9 subunits are the most frequently preferred Gγ subtypes in previous G protein biosensor research, these proteins were selected and N-terminally tagged with LSS-mKATE2.

To identify the optimal donor–acceptor pairs, each of the Gα-NLuc variants was co-transfected in HEK293T.WT cells along with one of the Gγ-LSS-mKATE2(N) subtypes and a GPCR for which the associated G protein coupling is well-established (further referred to as a model receptor). These model receptors were the human β2-adrenergic receptor (ADRB2; Gα_Ss/L_), Thromboxane A2 receptor (TBX; Gα_q/12/13/15_) and Histamine 3 receptor (H3R; Gα_i1-3/OA/OB_) [8]. Forty-eight hours post transfection, the donor substrate (Vivazine, Promega) was added, and after substrate stabilization, cells were stimulated with the appropriate agonist. BRET responses were continuously measured, and the Neg AUC was used as the readout for G protein activation.

**Figure 3:**
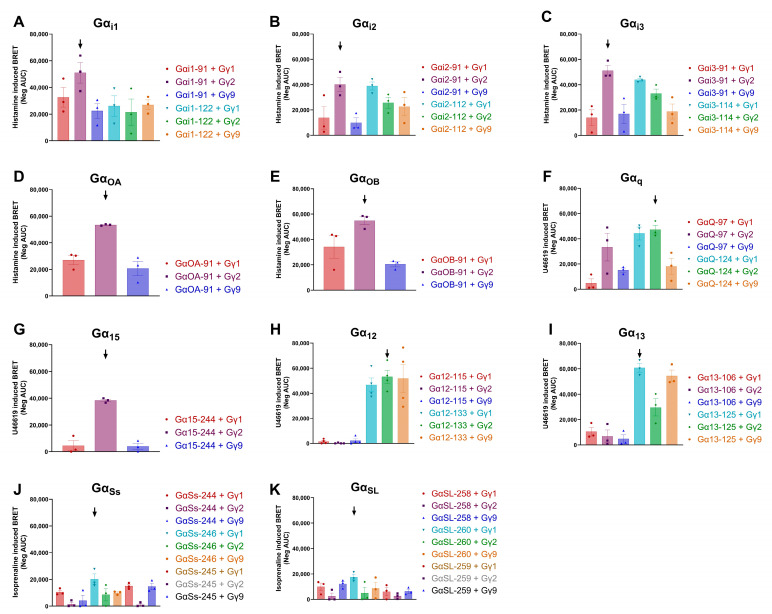
Optimal donor–acceptor pair determination for each Gα subunit. Cells co-expressing one Gα subunit (Gα_i1,-i2, -i3, -OA, -OB,-q, -15,-12,-13,-Ss,-SL_; panel (**A**–**K**), respectively) fused with NLuc (see legend for AA number of insertion place) along with one of the three N-terminally tagged Gγ subunits and a model receptor (β2-adrenergic receptor (ADRB2), Gα_Ss/L_; Thromboxane A2 receptor (TBX), Gα_q/12/13/15_ and Histamine 3 receptor (H3R), Gα_i1-3/OA/OB_), respectively, were stimulated with the appropriate agonist at a single concentration (1 µM U46619 (TBX; Gα_q/12/13/15_), 1 µM histamine (H3R; Gα_i1-3/OA/OB_) or 10 µM isoprenaline (ADRB2; Gα_Ss/L_). For each transfection, the BRET responses were measured, and area under the curve (AUC) was taken as readout. For each donor–acceptor plasmid combination, three to four independent transfections were performed, and the combination that generated the largest mean neg AUC was taken as the optimal pair (indicated with an arrow).

When co-transfected cells were stimulated with either 1 µM U46619 (TBX; Gα_q/12/13/15_) or 1 µM histamine (H3R; Gα_i1-3/OA/OB_), at least one of the donor–acceptor pairs showed clear BRET responses (Figure 3A–I). In contrast, when ADRB2-transfected cells were stimulated with 10 µM isoprenaline (ADRB2; Gα_Ss/L_), no significant BRET response compared to the vehicle response was detected (Figure S2). To overcome this lack of signal observed for Gα_Ss/L_, we performed an amino acid sequence comparison between Gα_15_ and Gα_Ss/L_ (Figure S3), based on which we identified three new possible donor insertion sites within the switch III region. Both for Gα_Ss_ and Gα_SL_, these new Gα_Ss/L_-NLuc variants rendered a significant BRET response upon the stimulation of ADRB2-expressing cells with 10 µM isoprenaline (Figure 3J,K). The donor–acceptor pair generating the highest averaged Neg AUC, based on three to four independent transfection experiments, was considered to be the most optimal plasmid pair for the corresponding G protein subtype (Figure 3). Similar protein expression of the differential donor and acceptor proteins was ensured by using flow cytometry and the initial bioluminescent donor emission of the model biosensor pair (Figure S4). An overview of the selected donor–acceptor pairs, which together make up the REGA-SIGN G protein biosensor panel, is presented in Table 2.

### 3.4. Validating the Biological Activity of the REGA-SIGN NLuc-Tagged Gα Proteins

**Figure 4:**
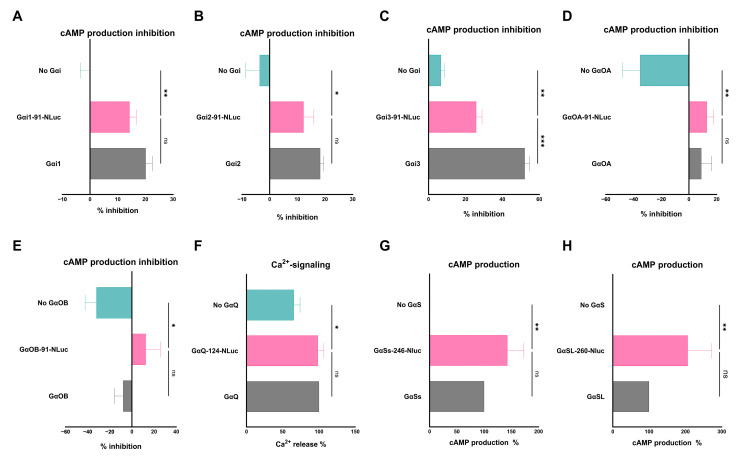
Rescue experiments validating the retained biological activity of NLuc-tagged Gα_i/o_, Gα_q_ and Gα_Ss/SL_ donor plasmids. (**A**–**E**) Rescue experiments where Histamine-mediated inhibition of forskolin-induced cyclic adenosine monophosphate (cAMP) production is measured. HEK293A Gα_i/o_ KO cells were co-transfected with Histamine 3 receptor (H3R), pGloSensor-22F and either NLuc-tagged Gα_i/o_, untagged Gα_i/o_ or transfection carrier DNA (No Gα_i/o_, NC). (**F**) Rescue experiment where U46619-mediated Ca^2+^ mobilization is measured. HEK293A Gα_q_ KO cells were co-transfected with Thromboxane A2 receptor (TBX) and either NLuc-tagged Gα_q_, untagged Gα_q_ or transfection carrier DNA (No Gα_q_, NC). (**G**,**H**) Rescue experiment where isoprenaline-mediated cAMP production is measured. HEK293A Gα_S_ KO cells were co-transfected with β2-adrenergic receptor (ADRB2), pGloSensor-22F and either NLuc-tagged Gα_S_, untagged Gα_S_ or transfection carrier DNA (No Gα_S_, NC). All data (**A**–**F**) show the mean ± SEM of three independent experiments or (**G**,**H**) normalized mean ± SEM of three independent experiments with the untagged Gα activity being set at 100% and the NC at 0%, respectively. One-way ANOVA followed by a Dunnett multiple-comparison test was able to show significant (* *p* < 0.05; ** *p* < 0.01, *** *p* < 0.001) and differences between the NC and the tagged Gα subunit, whereby no significant differences (ns) were found between the activity of tagged and untagged Gα subunits.

To ensure the retained biological activity of the selected Gα-NLuc variants, several rescue experiments were performed. For the NLuc-tagged Gα_Ss/L_, Gα_i/o_ and Gα_q_, HEK293 cells devoid of the relevant Gα protein (G protein knock-out (KO) cells, Table 1) were transfected with the appropriate model receptor and either untagged wildtype Gα protein, NLuc-tagged Gα protein or transfection carrier DNA. Afterward, G protein activity was assessed using relevant second-messenger assays, measuring cAMP production (Gα_Ss/L_), inhibition thereof (Gα_i/o_) or Ca^2+^ mobilization (Gα_q_), respectively. No significant (ns) differences were found between the activity of tagged and untagged Gα subunits, with the exception of Gα_i3_ where the tagged protein was less potent compared to its wild-type variant. However, the tagged Gα_i3_ subunit still showed significant biological activity compared to the carrier DNA control (NC) (Figure 4). These experiments demonstrated that the donor-tagged Gα subunits were still able to rescue cAMP production (Gα_Ss/L_), cAMP signaling inhibition (Gα_i/o_) or Ca^2+^ mobilization (Gα_q_).

### 3.5. Validation of REGA-SIGN Signal Specificity

**Figure 5:**
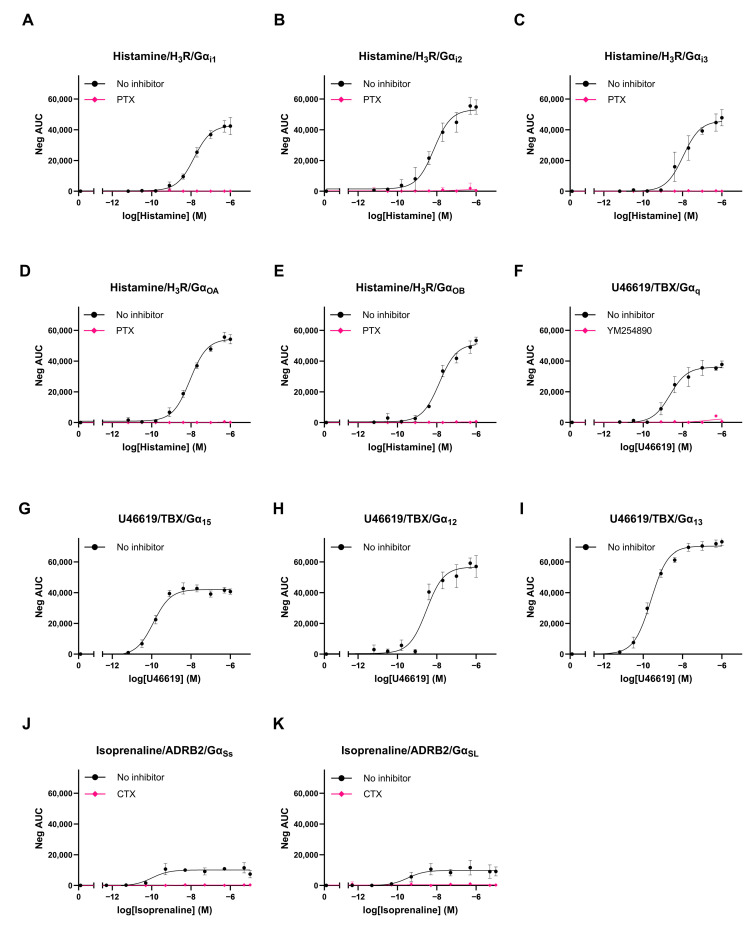
Dose-dependent BRET responses upon agonist stimulation and G protein inhibition. (**A**–**E**) HEK293T.WT cells co-transfected with NLuc-tagged Gα_i/o_ subunits, LSS-mKATE2-tagged Gγ2 subunit and the histamine 3 receptor (H3R) were stimulated with histamine in absence (black) or presence (pink) of Pertussis toxin (50 ng/mL). (**F**–**H**) HEK293T.WT cells co-transfected with NLuc-tagged Gα_q,15,12_ subunits, LSS-mKATE2-tagged Gγ2 subunit and the Thromboxane A2 receptor (TBX). Cells were stimulated with U46619 (1 µM) in absence (black) or presence (pink) of YM254890 (2 µM). (**I**) HEK293T.WT cells co-expressing NLuc-tagged Gα_13_ subunits, LSS-mKATE2-tagged Gγ1 subunit and the TBX receptor. Cells were stimulated with U46619 (1 µM). (**J**,**K**): HEK293T.WT cells co-expressing NLuc-tagged Gα_Ss/L_ subunits, LSS-mKATE2-tagged Gγ1 subunit and the β2-adrenergic receptor (ADRB2). Cells were stimulated with Isoprenaline (10 µM) in absence (black) or presence (pink) of Cholera toxin (CTX, 10 µM). The data represent the mean AUC ± SEM of three to four independent experiments. AUC was calculated based on the NCcorrected BRET ratios which were measured for 25 min after receptor stimulation. Dose–response curves were fitted to *log(agonist)* vs. *response model* in GraphPadV9.3.1.

The specificity of the obtained BRET signals was investigated by performing dose–response measurements in the presence or absence of established G protein inhibitors. HEK293T.WT cells were transiently co-transfected with a REGA-SIGN biosensor donor–acceptor pair and the appropriate model receptor. All G protein biosensors showed dose-dependent BRET responses following ligand-induced receptor activation (Figure 5). This dose dependency proved the ability of the tagged G proteins to rearrange and dissociate upon the stimulation of their model receptor. Furthermore, responses could be completely abolished by pre-incubating the cells with established G protein inhibitors (i.e., Pertussis toxin (PTX), Cholera toxin (CTX) or YM254890 for the inhibition of Gα_i/o_, Gα_Ss/L_ and Gα_q_, respectively) further proving their signal specificity (Figure 5). Unfortunately, to our knowledge, no Gα_12/13_ and Gα_15_ inhibitors exist. However, using donor-saturation assays (DSAs) as described by Promega [25] we could also validate their signal specificity (Figure S5).

### 3.6. Head-to-Head Comparison between EGFP and LSS-mKATE2

**Figure 6:**
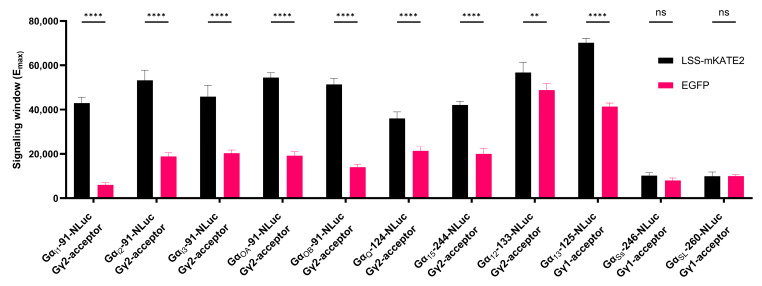
Head-to-head comparison of E_max_ between LSS-mKATE2 and EGFP. HEK293T.WT cells co-transfected with a Gα-NLuc variant, the appropriate Gγ subtype (Table 2) tagged with either LSS-mKATE2 or EGFP and the relevant model receptor were stimulated with a 1:3 dilution series of Histamine (1 µM), U46619 (1 µM) or Isoprenaline (10 µM), respectively. BRET ratios were measured for 25 min after receptor stimulation. Subsequently, the dose–response curves were fitted to *log(agonist)* vs. *response—Find ECanything model* in GraphPad V9.3.1 (GraphPad Software, San Diego, CA, USA) whereafter the calculated top values were taken as E_max_. Using one-way ANOVA followed by a Dunnett multiple-comparison test, significant differences (** *p* < 0.01 **** *p* < 0.0001) between the E_max_ of EGFP and LSS-mKATE2 were found for all Gα subunits, except for Gα_S_ subunits, where no significant difference (ns) was found. The data represents the mean E_max_ ± SEM of three independent experiments.

The choice of LSS-mKATE2 (λ_em_:605 nm) as an acceptor fluorophore in the REGA-SIGN biosensor package was to increase the spectral separation between donor (NLuc, (λ_em_:460 nm) and acceptor emission spectra compared to the commonly used EGFP (λ_em_:507 nm) or equivalent yellow- or green-shifted acceptor proteins (Figure 1A). Theoretically, this should significantly improve the signal-to-noise ratio and enhance the signal window, potentially leading to more sensitive biosensors. To evaluate the effect of LSS-mKATE2 vs. EGFP as acceptor fluorophores, a direct head-to-head comparison was performed. For this, HEK293T.WT cells were transiently co-transfected with the optimal Gα-NLuc variant, the appropriate model receptor and the preferred Gγ subtype tagged with either LSS-mKATE2 or EGFP, respectively. Using the E_max_ values, calculated by the non-linear curve fitting of the dose-dependent responses (Figure S6), an indication of the signal window of the different biosensors can be obtained. Upon the comparison of these E_max_ values, significant improvement in the signaling window and consequently improved sensitivity across all of the biosensors can be seen, with the exception of the Gα_S_ family for which both EGFP or LSS-mKATE2 biosensor plasmids showed similar sensitivity (Figure 6). Similar protein expression levels were ensured as exemplified by comparing the donor luminescent signal of the model G protein biosensor Gα_i1_-91-Nluc and newly developed biosensor Gα_Ss_-246-Nluc when co-transfected with, respectively, Gγ2 and Gγ1 tagged with either LSS-mKATE2 or EGFP (Figure S7).

## 4. Discussion

In this study, we developed and validated a novel set of G protein biosensors, designated as REGA-SIGN, covering the entire family of G proteins and allowing for the kinetic investigation of G protein activation. NLuc and LSS-mKATE2 were chosen as the respective donor and acceptor molecules. We were interested in evaluating their application in a G protein biosensor approach given their large spectral separation and potential compatibility [16], despite LSS-mKATE2 having lower brightness and a longer protein maturation time compared to other fluorescent proteins [28,31]. Regardless of these disadvantages, our study shows that choosing LSS-mKATE2 as an acceptor fluorophore enables robust and specific BRET signals with limited to no contamination of donor emission in the energy acceptor filter (Figure 1).

Given that the co-transfection of multiple plasmids could induce more variation in cell-to-cell protein expression levels due to translation resource competition, we aimed to set up a sensitive and reproducible G protein biosensor approach with a minimal amount of co-transfected plasmids [32]. In 2021, Schihada et al. [11] also tackled this issue by creating a multi-cistronic plasmid whereby only one transfection is required since all relevant biosensor components are combined within a single plasmid. However, using this approach does not allow one to optimize donor–acceptor ratios and bears a risk of incomplete cleavage of the translated polypeptide [32]. Furthermore, data of a recent biosensor study [30] confirmed that the co-transfection of untagged Gβ1 is not strictly necessary to generate functional heterotrimeric G protein complexes. Unlike the stoichiometric expression of the three G protein subunits which is often aimed for [8], we decided to evaluate a biosensor set-up consisting of only two plasmids (i.e., Gα-NLuc and Gγ-LSS-mKATE2). However, to experimentally evaluate the effect of leaving out Gβ1, we compared the biosensor readout for one particular donor–acceptor combination in the absence or presence of Gβ1. Interestingly, including Gβ1 during the transfection did not affect the observed potency of receptor activation, in line with recent findings [30], but did lower the E_max_ (Figure 2). Currently, we do not have any conclusive mechanistic insight explaining this observation. To our knowledge, little is known about the relative expression levels of G protein subunits in vivo and how this is affected by transfection procedures. It might be hypothesized that the amount of functional heterotrimeric Gαβγ complexes formed depends on the G protein subunit being present in the least amount. In our assay set-up, this would imply that the amount of the functional heterotrimeric G protein complex would be determined by the relative abundance of endogenous, physiologically relevant levels of Gβ1. Co-transfecting the tagged donor and acceptor plasmids makes sure that these Gβ1 subunits preferentially interact with tagged donor and acceptor proteins rather than with their untagged Gα and Gγ counterparts. Although additional transfection of Gβ1 would give rise to increased levels of Gαβγ complexes this may not necessarily lead to better responses (and in our case, even to a lower response). Here, one can hypothesize that likely not all G protein complexes will be involved in receptor activation. If only a minor part of the Gαβγ complexes becomes activated, the relative amount of Gαβγ that remains unaffected upon receptor stimulation (and thus does not dissociate) will probably affect the signal window. Altogether, our data did confirm that co-transfection with a Gβ1-encoding plasmid could be omitted (Figure 2), and Gβ1 plasmids were therefore not further included into the REGA-SIGN biosensor package.

To achieve optimal sensitivity for the eleven different biosensors (Gα_i1_, Gα_i2_, Gα_i3_, Gα_OA_, Gα_OB_, Gα_Ss_, Gα_SL_, Gα_12_, Gα_13_, Gα_q_ and Gα_15_) included in REGA-SIGN donor and acceptor molecules should be in their most optimal relative position to each other. Based on previous research, we therefore generated a whole set of G protein biosensor plasmids. For all subtypes of Gα proteins, with the exception of Gα_Ss/SL_, one of the selected donor insertion sites [8,9,11,17] resulted in a donor–acceptor pair able to detect G protein activation upon the agonist stimulation of the model GPCRs (Figure 3). With the exception of Gα_S_ and Gα_i3_, all the donor (NLuc) insertion sites found to be the optimal ones in our experimental design corresponded to the insertion sites designated by TRUPATH [8]. The alternative donor insertion sites previously used in a tricistronic NanoBRET-based G protein biosensor [11] were not the most ideal in our setting. This was interesting since REGA-SIGN and the tricistronic biosensor both used NLuc as a donor molecule while TRUPATH is a thoroughly optimized eBRET2-based G protein biosensor using RLuc8. However, in eight out of the eleven REGA-SIGN donor–acceptor pairs, Gγ2 was identified as the optimal acceptor protein, which did correspond to the tricistronic biosensor using a YFP acceptor fluorophore. On the other hand, TRUPATH, using GFP2 as a fluorescent protein, never identified Gγ2 as the optimal acceptor protein [8,11]. These contrasting results show the importance of the optimization process and may be explained by the very nature of the BRET assay. The biosensor donor–acceptor pairs are chosen because of their optimal BRET efficiency. Consequently, these biosensors reflect the Gα- and Gγ-protein pair with the most optimal heterotrimeric complex stability and relative orientation of the Gα and Gγ subunits, generating the most pronounced BRET signal. For REGA-SIGN, we chose different Gα- and Gγ-protein tags and did not perform co-transfection with a Gβ-encoding expression plasmid. These choices may result in subtle alterations in the conformation of the heterotrimeric G protein complex and thus differences in optimal donor–acceptor pairs compared to the other G protein biosensor approaches.

The optimal REGA-SIGN biosensor pairs were further validated by means of rescue experiments and BRET signaling inhibition with specific G protein inhibitors. All biosensor pairs interacted specifically with each other (Figure 5), and for Gα_i/o_, Gα_Ss/L_ and Gα_q_, we proved that integrating NLuc into the Gα protein did not significantly hamper its biological signaling activity (Figure 4). These results suggest that the biologically relevant conformation of the G protein is retained, and therefore, meaningful data can be extracted when using these tagged G proteins. For Gα_i1_ and Gα_q_, previous studies [8,33] supported this statement by performing similar rescue experiments, while, to our knowledge, for the other Gα variants, no data regarding the biological activity of donor-tagged Gα proteins are available. In the case of Gα_15_ and Gα_12/13_, we were unable to perform rescue experiments since no corresponding G protein KO cells and/or downstream signaling readout specific for the G protein were available, respectively. While previous studies were able to perform rescue experiments showing unhampered biological activity for Gα_15_ tagged at the equivalent insertion place [8], for Gα_12_ and Gα_13_, it has never been shown. However, all the Gα-NLuc variants, including Gα_12/13_ and Gα_15_, did show dose-dependent responses upon ligand stimulation (Figure 5) proving their ability to rearrange upon receptor activation. Therefore, the above-mentioned results confirm that REGA-SIGN biosensors are able to specifically detect G protein activity by measuring NanoBRET signals without altering their biological signaling activity.

We hypothesized that pairing NLuc with LSS-mKATE2 and using Vivazine as a stable donor substrate might result in advantages over other existing methods. The primary advantages would be the ability to perform kinetic readouts and improved biosensor sensitivity. Since the existing methods using RLuc8 or NLuc are always paired with a green- or yellow-shifted fluorophore, REGA-SIGN’s increased sensitivity would come forward from the improved spectral separation between the donor and acceptor emission peaks. To test this hypothesis, we implemented a head-to-head comparison between the NLuc paired with either LSS-mKATE2 or EGFP which results in a spectral separation of, respectively, 145 nm or 47 nm. Here, the results showed that using the red-shifted fluorophore indeed improved the sensitivity significantly across all biosensor pairs, with the exception of Gα_S_ where it remained similar. Of note, the signal window achieved by these Gα_Ss_ and Gα_SL_ subunits was smaller compared to the other LSS-mKATE2 biosensors. Given that a lower signal window was also present in the EGFP couple and other previously established BRET-based Gα_s_-activity biosensors, it is likely to be an inherent feature of Gα_S_ heterotrimer rearrangement [8,11,17]. Overall, the results confirm that combining NLuc with LSS-mKATE2 is advantageous over pairing it with EGFP or an equivalent green- or yellow-shifted fluorophore. Obviously, although this head-to-head comparison clearly points at the important choice of acceptor fluorophore, other experimental parameters (e.g., type of donor, donor-insertion site, effect of co-expressing Gβ) may also affect the overall performance of G protein biosensors. Nevertheless, using the optimized LSS-mKATE2 protocol, REGA-SIGN showed to be a kinetic G protein biosensor with enhanced sensitivity.

## 5. Conclusions

REGA-SIGN was optimized and validated as a new G protein biosensor package. As evidenced by the stable donor luminescent signal over time, it is one of the few G protein BRET biosensors that can be used in a kinetic reading method. Unlike previous G protein BRET-based biosensors, it does not rely on the co-transfection of an untagged β1 protein and uses a red-shifted fluorophore, resulting in a kinetic G protein biosensor with enhanced sensitivity. These characteristics make REGA-SIGN particularly useful as a screening tool to provide kinetic information about G protein signaling at a single-pathway resolution. This knowledge will advance the understanding of, for example, biased G protein signaling, which could lead to new therapeutic approaches to combat GPCR-related pathologies.

## Figures and Tables

**Table 1:** Overview of G protein KO cell lines.

Cell Line	Knocked-Out Gene
HEK293-ΔGα_S_	GNAS/GNAL
HEK293-ΔGα_i_	GNAI1, GNAI2, GNAI3, GNAO1, GNAZ, GNAT1, GNAT2
HEK293-ΔGα_q_	GNAQ, GNA11

**Table 2:** Donor–acceptor plasmid pairs used in REGA-SIGN. Optimal donor–acceptor pairs (i.e., donor–acceptor combinations that yield the highest neg AUC upon stimulation of model GPCRs) are shown. NLuc cDNA is integrated between the two amino acids whereby the number indicates the position of the last amino acid flanking the N-terminus of the NLuc insert. REGA-SIGN donor and acceptor plasmids are available on Addgene.

Gα Protein Plasmid	NLuc Position	Gγ-LSS-mKATE2(N)
Gα_i1_	L(91)K	Gγ2
Gα_i2_	L(91)Q	Gγ2
Gα_i3_	L(91)K	Gγ2
Gα_OA_	L(91)G	Gγ2
Gα_OB_	L(91)G	Gγ2
Gα_q_	F(124)E	Gγ2
Gα_12_	A(133)F	Gγ2
Gα_13_	F(125)D	Gγ1
Gα_15_	E(244)N	Gγ2
Gα_Ss_	D(246)N	Gγ1
Gα_SL_	D(260)N	Gγ1

## Data Availability

Data are contained within the article or supplementary material.

## Supplementary Materials

The following supporting information can be downloaded at https://www.mdpi.com/article/10.3390/bios13080767/s1, Figure S1: Graphical representation of BRET data analysis; Figure S2: Initial Gα_S_ donor insertion site analysis; Figure S3: Gα_15,Ss,SL_ subunit protein sequence alignment; Figure S4: Expression level comparison between Gα_i1_ donor and acceptor pair combinations. Figure S5: Donor saturation assays (DSAs) to confirm BRET-signal specificity. Figure S6: Head-to-head comparison of the dose-dependent BRET responses upon agonist stimulation between LSS-mKATE2 and EGFP acceptor. Figure S7: Donor protein expression comparison for Gαi1 and GαSs between co-transfection with Gγ tagged with LSS-mKATE2 or EGFP.
