# Supplementary material for: REGA-SIGN: Development of a Novel Set of NanoBRET-Based G Protein Biosensors
Source: Biosensors (Basel). 2023 Jul 28;13(8):767. doi: 10.3390/bios13080767 (PMC10452170; doi:10.3390/bios13080767)
Supplement: Supplementary file 1 [file biosensors-13-00767-s001.zip › biosensors-2370139-supplementary.pdf]

biosensors

MDPI

Supplementary data

REGA-SIGN: Development of a Novel Set of NanoBRET-Based G Protein Biosensors

Katrijn Boon, Nathan Vanalken, Eef Meyen, Dominique Schols and Tom Van Loy *

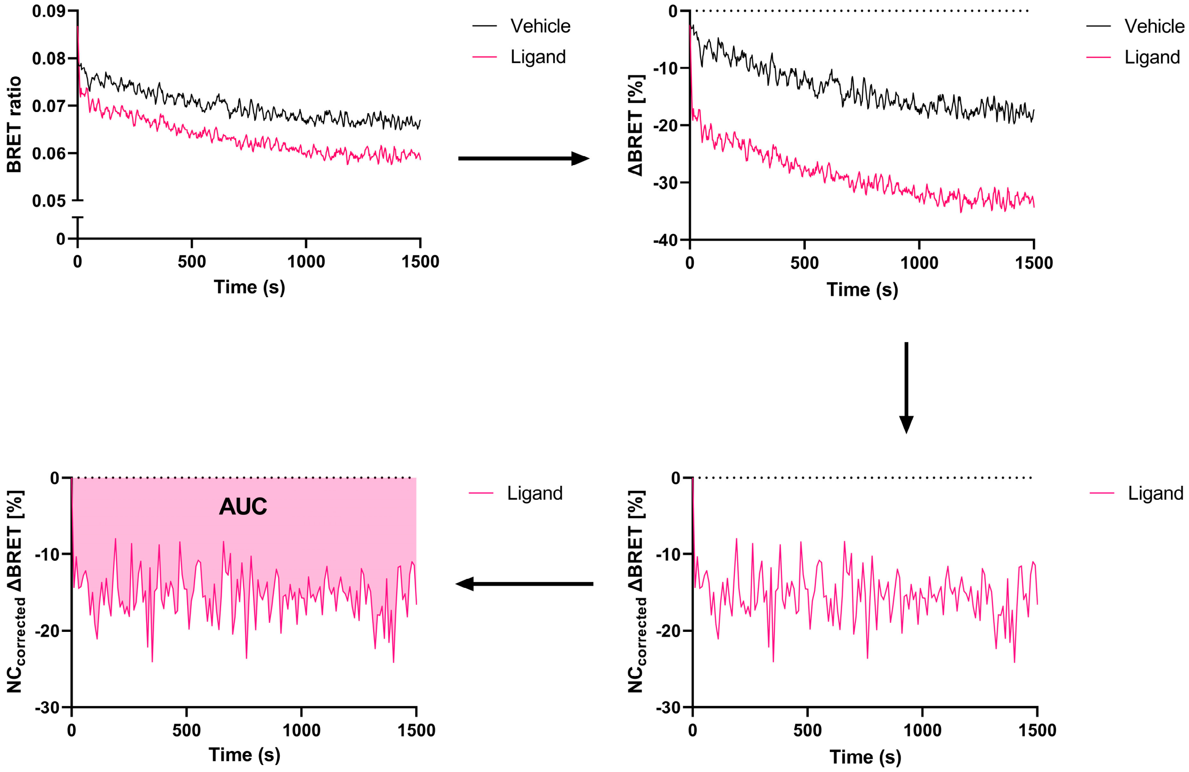

Figure S1: Graphical representation of BRET data analysis (A) BRET ratios were calculated by taking the ratio of LSS-mKATE2 acceptor emission (615nm) to NLuc donor emission (440 – 480 nm). The basal BRET ratio ($Ratio_{basal}$) was then defined as the mean BRET ratio of the five consecutive readings prior to ligand addition. (B) To quantify ligand-induced changes, ΔBRET was calculated for each well as a % difference to baseline. (C) ΔBRET values were background corrected by subtracting the negative control averaged ΔBRET. (D) The negative Area under the curve (neg AUC) was used as the readout for G protein activation**.**

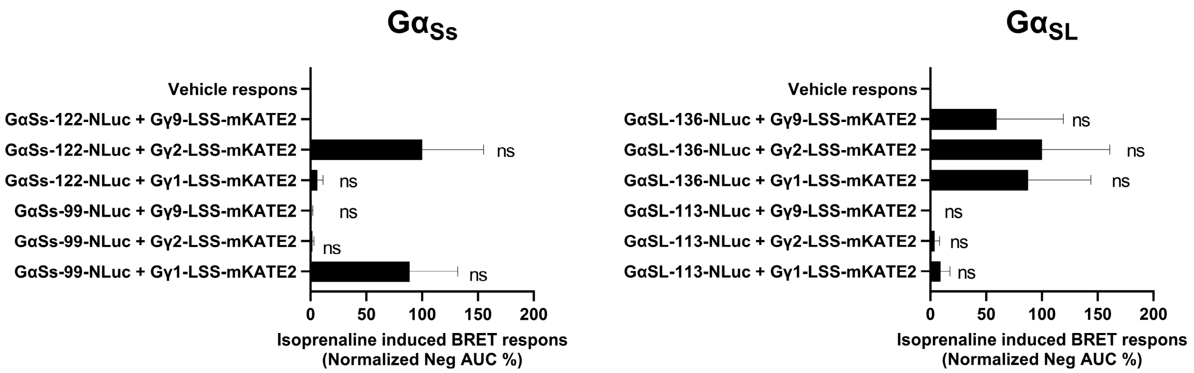

Figure S2: Initial Gαs donor insertion site analysis Cells co-expressing one Gα subunit carrying NLuc (see legend for AA number of insertion place) along with one of the three N-terminally tagged Gγ subunits and the β2-adrenergic receptor (ADRB2) were stimulated with 10 μM isoprenaline. For each transfection, the area under the curve (AUC) was normalized and AUC of the eventual superior donor acceptor pair was chosen as 100%. Data represents the mean of three independent transfections ± SEM. One-way ANOVA followed by a Dunnett's test was unable to show significant differences (ns) between the vehicle response and the detected BRET response of the first set of Gα_{Ss} and Gα_{Ss} biosensors.

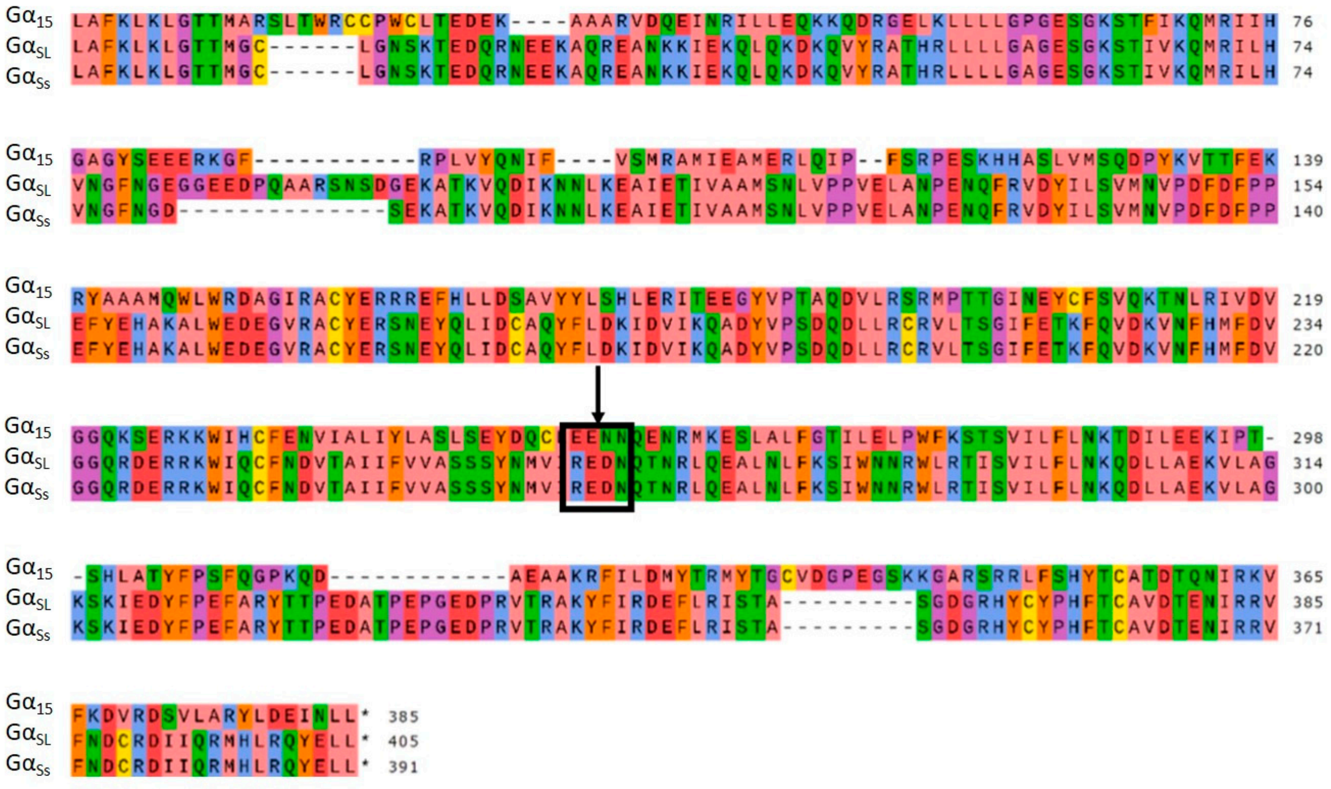

Figure S3: G$\alpha_{15,Ss,SL}$ subunit protein sequence alignment. Gα protein sequence alignment was executed using Clustal Omega. Amino acids (AA) are highlighted based on properties and conservation (Clustal X) in pink/blue, red, green, yellow, purple, and orange for aliphatic/hydrophobic, positive, negative, cysteine, conformationally special, and aromatic AA, respectively. The black box indicates a conserved region within the switch III region selected to evaluate three potential insertion sites: R(258)E, E(259)D, and D(260)N for Gα_{SL} and R(244)E, E(245)D and D(246)N for Gα_{Ss}. The black arrow indicates the optimal insertion site of NLuc in the Gα_{15} subunit, as described recently¨[8].

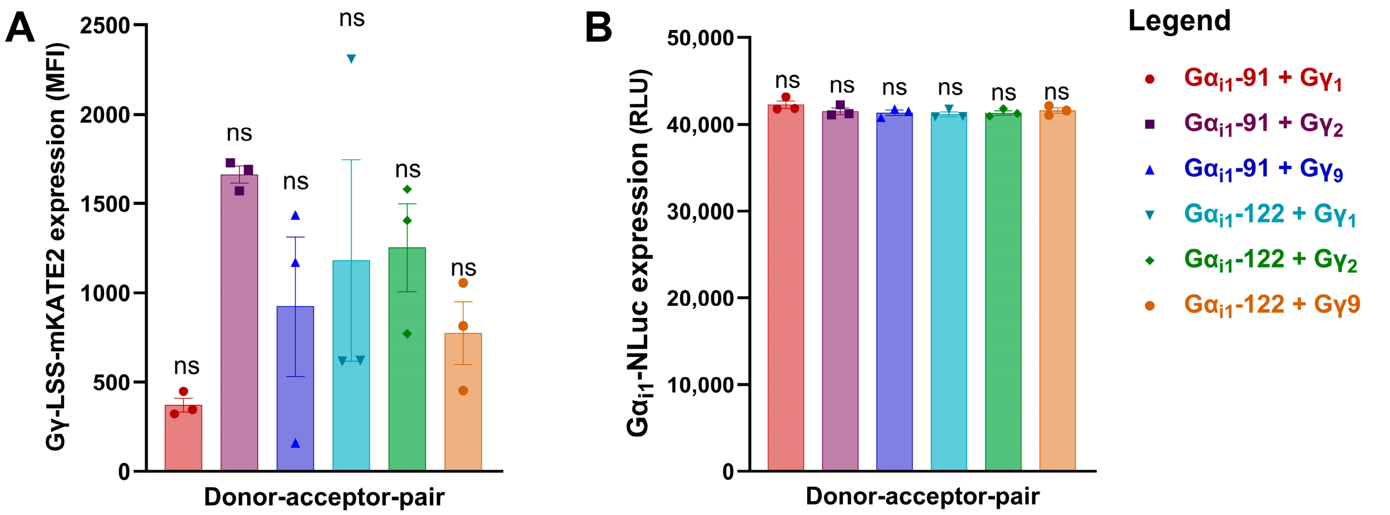

Figure S4: Expression level comparison between $G\alpha_{i1}$ donor and acceptor pair combinations. Cells co-expressing one $G\alpha_{i1}$ subunit carrying NLuc (see legend for AA number of insertion place) along with one of the three N-terminally tagged Gγ-subunits and the Histamine 3 receptor (H3R) were analyzed. (A) For each transfection total LSS-mKATE2 fluorescence was determined by flow cytometry as a surrogate for the acceptor expression levels. (B) Luminescent donor emission signals, were used as a surrogate for the donor expression levels. Data represents the means of three independent transfections ± SEM. Data was tested by using a one-way ANOVA, followed by Tukey's multiple comparisons in GraphPadV9.3.1. For convenience, only the statistical comparisons between the chosen optimal donor-acceptor pairs are presented on the figures, which were all not significantly different (ns).

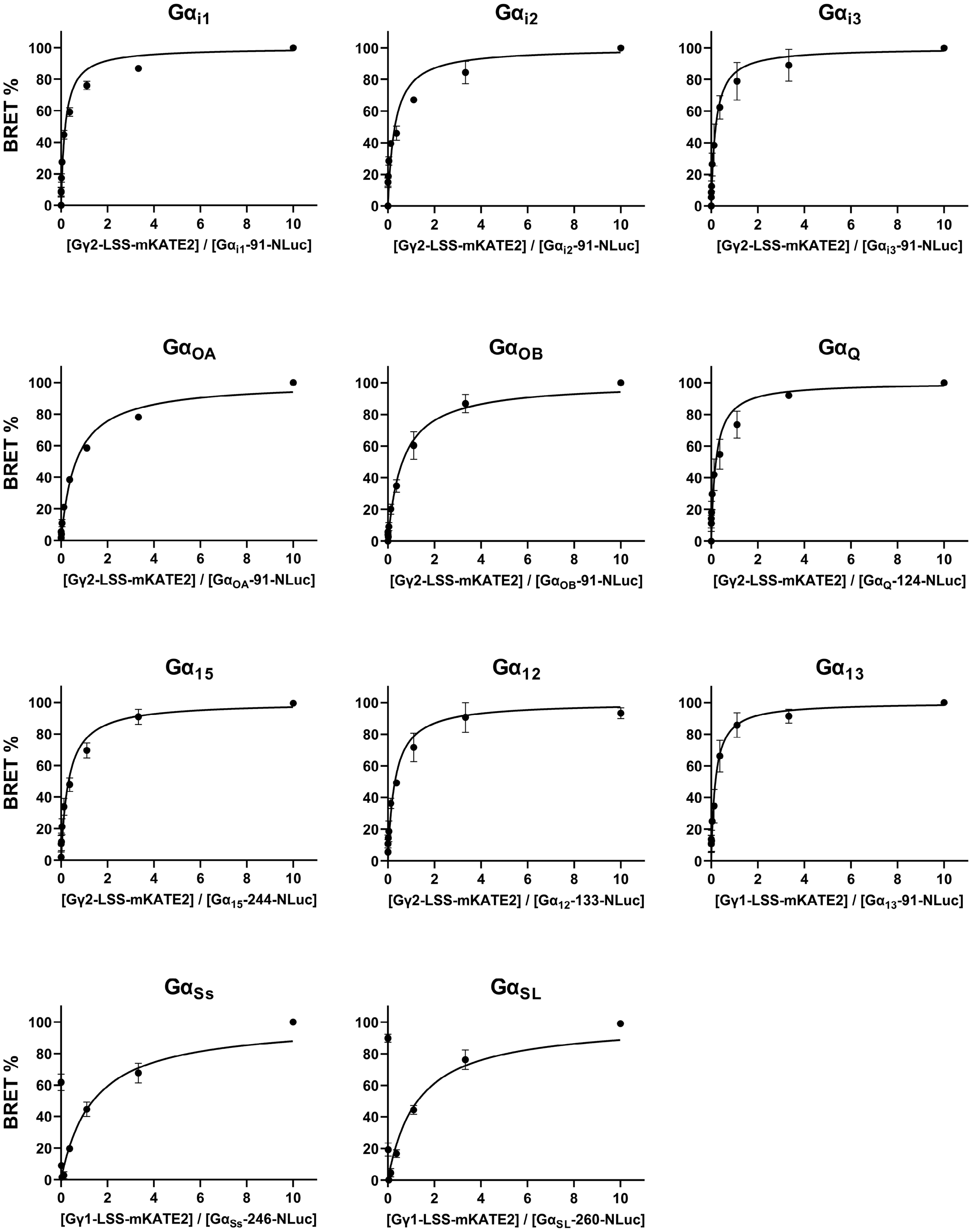

Figure S5. Donor saturation assays (DSAs) to confirm BRET-signal specificity. Cells were co-transfected with a fixed amount of Gα donor plasmid (100 ng) and increasing amounts of Gγ acceptor plasmid (up to 1000 ng). This led to an increase of the basal BRET signal with hyperbolic curves reaching a plateau in function of increasing acceptor plasmid, indicating specific BRET signals. The BRET ratio at a 10:1 acceptor-to-donor ratio was always set as 100%. Data represents the mean ± SEM of three to six repeats. Curves were fitted to *[Agonist] vs. normalized response model* in GraphPadV9.3.1

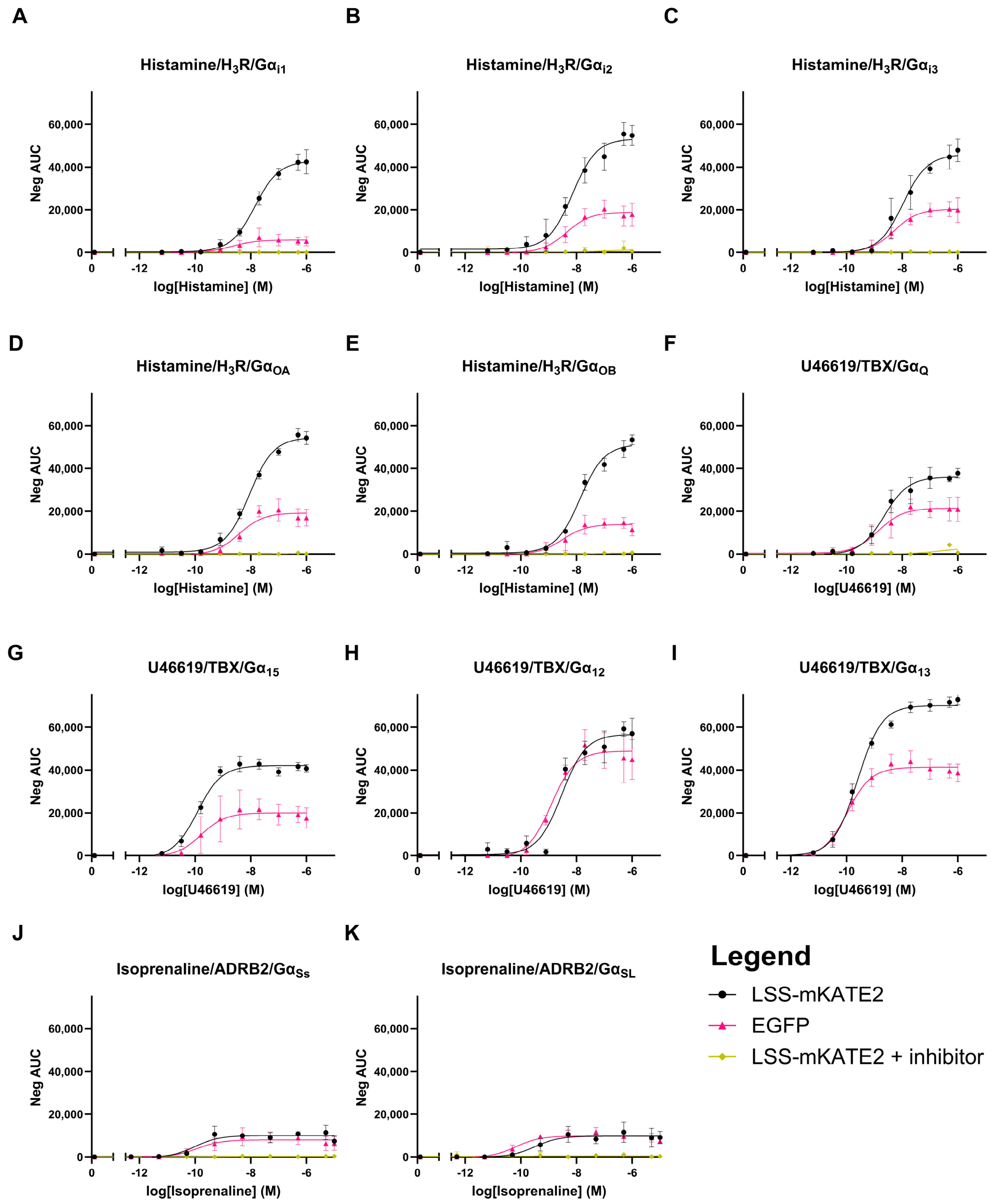

Figure S6: Head-to-head comparison of the dose-dependent BRET responses upon agonist stimulation between LSS-mKATE2 and EGFP acceptor (A-E) HEK293T.WT cells co-transfected with NLuc-tagged G$\alpha_{i/o}$ subunits, LSS-mKATE2 or EGFP-tagged Gγ2 subunit and H3R were stimulated with histamine in absence (black) or presence (pink) of Pertussis toxin (50 ng/mL). (F-H) HEK293T.WT cells co-transfected with NLuc-tagged G$\alpha_{q,15,12}$ subunits, LSS-mKATE2 or EGFP-tagged Gγ2 subunit and TBX receptor. Cells were stimulated with U46619 (1µM) in absence (black) or presence (pink) of YM254890 (2µM). (I) HEK293T.WT cells co-expressing NLuc-tagged Gα_{13} subunits, LSS-mKATE2- or EGFP-tagged Gγ1 subunit and TBX receptor. Cells were stimulated with U46619 (1µM). (J-K) HEK293T.WT cells co-expressing NLuc-tagged G$\alpha_{Ss/L}$ subunits, LSS-mKATE2- or EGFP-tagged Gγ1 subunit and ADRB2. Cells were stimulated with Isoprenaline (10µM) in absence (black) or presence (pink) of Cholera Toxin (CTX, 10µM). Data represents the mean AUC ± SEM of three to four independent experiments. AUC was calculated based on the NC$_{corrected}$ BRET ratios measured for 25min after receptor stimulation. Dose-response curves were fitted to *log(agonist) vs. response—Find ECanything model* in GraphPad V9.3.1 (GraphPad Software, San Diego, CA, USA) whereafter the calculated top values were taken as E$_{max}$.

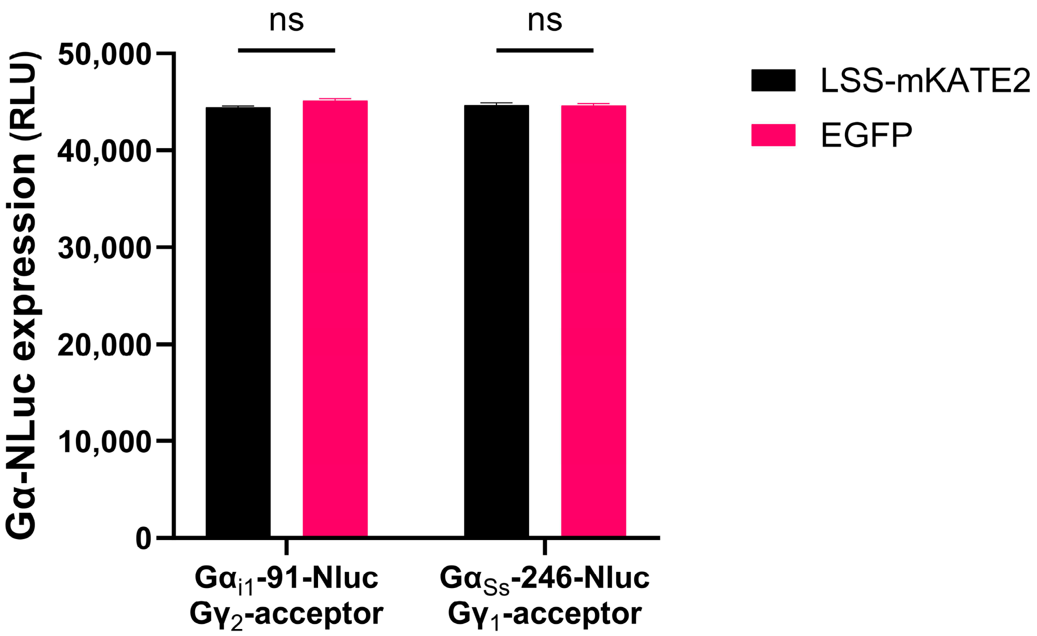

Figure S7: Donor protein expression comparison for Gαi1 and GαSs between co-transfection with Gγ tagged with LSS-mKATE2 or EGFP. HEK293T.WT cells co-transfected with respectively Gα$_{i1}$-91-NLuc or Gα$_{Ss}$-246-NLuc, LSS-mKATE2 or EGFP-tagged Gγ2 or Gγ1 subunit and the H3R or TBX receptor. Luminescent donor emission signals were used as a surrogate for the donor expression levels. Data represents the means of three independent transfections ± SEM. Data was tested by using a multiple T-test in GraphPadV9.3.1. No significant differences (ns) were found between the donor expression levels.
